# Global Guidance for Dyslipidaemia Management in Adults: A Scoping Review

**DOI:** 10.5334/gh.1506

**Published:** 2025-12-16

**Authors:** Andrew E. Moran, Ashish Krishna, Lawrence Mbuagbaw, Kouamivi M. Aboyibor, Rauell J. Santos

**Affiliations:** 1 Columbia University Irving Medical Center, New York, USA; 2 Resolve to Save Lives, New York, USA; 3 World Health Organization, Geneva, Switzerland; 4 McMaster University, Hamilton, Canada

**Keywords:** World Health Organisation, Lipid lowering therapy, Dyslipidaemia, Guidelines, Scoping Review

## Abstract

**Objective:**

Dyslipidaemia is a leading preventable cause of global cardiovascular disease (CVD) burden, responsible for over four million deaths each year (1). A scoping review took a worldwide perspective and assessed recent World Health Organization (WHO) guidance documents, other national or regional guidelines, and randomized controlled trial evidence supporting dyslipidaemia management best practices.

**Methods:**

Review of WHO guidance documents included aspects of dyslipidaemia management, but none provided a detailed and comprehensive approach. Of 11 non-WHO national or regional dyslipidaemia guidelines, nine met pre-defined inclusion criteria and were reviewed in depth. Structured electronic searches of MEDLINE found 27 systematic reviews of randomized clinical trials supporting dyslipidaemia management guideline priority topics.

**Findings:**

This scoping review found overall consistency in the recommendations of regional and national dyslipidaemia guideline recommendations. Guidelines varied in terms of approach to assessing patient CVD risk and recommendations to treat with non-statin lipid-lowering therapy (LLT). Robust randomized trial evidence supports that a dyslipidaemia management guideline focuses on priority areas including approach to patient selection for LLT efficacy and safety, selection of initial LLT drugs and dose intensity, timing of LLT monitoring, and LLT management in specific high-risk groups (familial hypercholesterolemia, diabetes, chronic kidney disease, HIV and other inflammatory diseases, and older adults). Few regional or national guidelines provided practical implementation recommendations or cost-effectiveness assessments; less clinical trial evidence was found for the priority topic of equitable treatment.

**Conclusion:**

Taking a global perspective, this scoping review describes the scope and depth of the current evidence base informing best practice management of dyslipidaemia for the primary and secondary prevention of CVD.

## Introduction

Cardiovascular disease (CVD) remains the leading preventable cause of death globally. Dyslipidaemia, particularly elevated total cholesterol (≥ 5.0 mmol/L or 190 mg/dL), is a significant risk factor for atherosclerotic cardiovascular diseases, such as coronary artery disease and stroke, and is estimated to cause 2.6 million preventable deaths annually (4% of all deaths) (2). Despite robust evidence supporting lipid-lowering therapies (LLTs) in both primary and secondary prevention, global treatment gaps persist: only 16% of patients with CVD receive statins for secondary prevention (3), and just 7% of eligible individuals receive statins for primary prevention (4).

Statins, the cornerstone of dyslipidaemia management, are widely available, safe, and cost-effective. Other LLTs (e.g., ezetimibe, PCSK9 inhibitors), when added to statins, provide combination LLT options, though cost and access to these agents vary significantly among countries and populations.

A global dyslipidaemia management guideline is needed. A clinical guideline provides clinicians ‘choice among different interventions or measures’ ‘to achieve the best health outcomes possible, individually or collectively’ (5). Existing clinical practice guidelines developed by national and regional bodies offer valuable guidance but are often tailored to specific health system capacities and may not be applicable across diverse global settings. The World Health Organisation (WHO) develops clinical guidelines whenever the Member States or other stakeholders ask for guidance. To date, the WHO has not published a dedicated global guideline for dyslipidaemia management in adults. A global guideline will need to synthesize recent evidence in lipidology and therapeutics, integrate advancements in CVD risk assessment, and focus on interventions that are feasible and impactful in primary health care settings, where most dyslipidaemia management is delivered.

This scoping review takes a global perspective and aims to map existing adult dyslipidaemia guidelines and supporting evidence, identify areas of consensus and variation, and inform the development of a global dyslipidaemia management guideline.

## Methods

This scoping review conformed to the PRISMA Extension for Scoping Reviews (PRISMA-ScR) reporting criteria (checklist, **Methods Appendix A**). Taking a global perspective, this review assessed 1) WHO dylipidaemia guidance documents published to date, 2) recent non-WHO national and regional dyslipidaemia management guidelines, and 3) availability of randomized, controlled trial evidence assessing LLT management priority topics.

### Selection of past WHO dyslipidaemia guidance documents

The WHO has provided clinical guidance on dyslipidaemia management as one component of disparate guidance documents, including formal technical packages, cardiovascular risk assessment tools, health care facility performance indicators, and medication access surveys and recommendations. A desk review of past WHO dyslipidaemia management guidance documents combined searches of the main and WHO regional websites using sequential Boolean search strings such as ‘dyslipidaemia AND guideline’, ‘lipid-lowering’, ‘statin’, ‘statin AND diabetes’, and ‘cardiovascular disease prevention’. This search was supplemented by key informant interviews of WHO staff participating in this review.

### Selection of past non-WHO dyslipidaemia management guidelines

Dyslipidaemia definitions are shown in **Appendix Table 1**. Inclusion criteria for non-WHO guidelines are listed in **Appendix Table 2**. The intent was to evaluate recent dyslipidaemia guidelines against a purpose-built evaluation metric. Regional and national guidelines were identified first by reviewing dyslipidaemia guidelines identified by selecting ‘cardiovascular disease policies’, then entering ‘dyslipid’ as a search term on the WHO noncommunicable diseases (NCD) document repository site (6), followed by purposeful ‘dyslipidaemia’ and ‘guideline’ MEDLINE keywords search for additional clinical guidelines and adding as keywords the names of world regions and most populous countries (Europe region, Asia Pacific region, the United States, China, India, Brazil, and Mexico). Only guidelines published in English, Spanish, or Portuguese were reviewed due to limited resources available for translation.

### Assessment for availability of systematic reviews of randomized controlled clinical trials

Two authors (AEM and AK) conducted structured searches of MEDLINE for systematic reviews supporting the development of a global dyslipidaemia management guideline (**Methods Appendix B, Table 2**). Searches of the MEDLINE electronic peer-reviewed literature repository used keywords and structured Medical Subject Headings (MeSH) terms. One author (AEM) selected systematic review articles based on title review; then two authors (AEM and AK) decided on the final inclusion based on a review of the article abstract and an assessment of inclusion criteria: systematic review of randomized controlled trials (RCTs) with hard CVD outcomes, synthesis using meta-analysis, and addressing dyslipidaemia management priority topics (LLT eligibility, LLT choice, LLT goals, LLT safety and effectiveness monitoring and follow up, and equity and cost-effectiveness, **Appendix Table 3**). A third reviewer (LM) served as a tie-breaker in case the original two reviewers disagreed regarding article inclusion. A ‘snowballing’ method identified additional relevant systematic review articles referenced by or referencing the included articles. Finally, the authors searched reference lists of UpToDate online medical encyclopaedia entries for ‘*Low-density lipoprotein cholesterol-lowering therapy in the primary prevention of cardiovascular disease*’ (7) and ‘*Management of low-density lipoprotein cholesterol (LDL-C) in the secondary prevention of cardiovascular disease*’ (8). The review was validated by checking that major systematic review articles published by the Cholesterol Treatment Trialists group were identified and included. Studies identified were evaluated for inclusion of outcomes data for specific primary prevention high-risk groups (e.g., familial hypercholesterolemia, diabetes, chronic kidney disease (CKD), HIV, or the elderly).

## Results

### Prior WHO dyslipidaemia definitions and clinical management guidance

The WHO defines raised cholesterol as total cholesterol ≥5.0 mmol/l or 190 mg/dl (2). This approximates a low-density lipoprotein cholesterol (LDL-C) of ≥3.5 mmol/l or 130 mg/dl (**Appendix Table 1**). Several past WHO guidance documents included aspects of dyslipidaemia management, including the WHO PEN Package (2000–2020), EMRO region guideline for dyslipidaemia management in people with diabetes (2006), the WHO CVD prevention guideline (2007), and the HEARTS ‘D’ Module (2020). These publications consistently recommended patient selection for statin LLT guided less by serum cholesterol level and more by overall estimated CVD risk.

The **2007 WHO Guideline on Prevention of CVD** (9) (‘Guidelines for assessment and management of cardiovascular risk’) recommended a CVD-risk guided approach to selecting patients for preventive therapies. Age- and sex-specific CVD risk assessment was calibrated for 14 distinct geographic regions in WHO/ISH risk assessment charts for primary prevention, excluding patients with existing CVD, left ventricular hypertrophy, severely high cholesterol (total cholesterol >= 8.0 mmol/L; 320 mg/dl, diabetes, or CKD). Along with other preventive treatments, the 2007 WHO guideline recommended diet and lifestyle interventions for all people without the high-risk features above and a 10-year CVD risk >= 10%. Statin treatment recommendation was definite for 10-year CVD risk >= 30% and recommended only if diet and lifestyle treatments did not reduce total cholesterol to <5.0 mmol/L or LDL cholesterol to <3.0 mmol/L among people with 10-year CVD risk of 20–30%.

This guideline did not provide recommendations for primary prevention pharmacologic LLT among people with moderately elevated cholesterol and 10-year CVD risk below 20%.

The **WHO Package of Essential Non-communicable (PEN) disease interventions** (10) was introduced in 2010, updated in 2013, and updated again, along with the WHO-HEARTS technical package, in 2020. The WHO PEN emphasized the use of 10-year CVD risk assessment charts, which integrated total cholesterol as a predictor where laboratory testing of lipid levels is available. Statin treatment was recommended for patients with 10-year CVD risk >= 20%. Like the 2007 CVD prevention guideline, the 2020 WHO PEN guideline recommended that the decision to initiate statin should be automatic (without 10-year CVD risk assessment) in patients with existing CVD, left ventricular hypertrophy, severely high cholesterol (total cholesterol >= 8.0 mmol/L; 320 mg/dl, diabetes, or CKD).

In 2019, the WHO translated data from the Emerging Risk Factors Collaboration into the updated **WHO 10-year CVD risk assessment charts** for use in primary care settings in 21 world regions (11,12). Global-level 2019 WHO risk equations were validated against external cohort studies. Considerable variability in 10-year CVD risk was found between regions for the same risk features, suggesting that the Member States should rely on their own region-specific 10-year CVD risk prediction algorithm. When the serum cholesterol number is available, it can be included as a variable in the risk assessment equation; however, the WHO non-laboratory risk assessment charts (not requiring serum cholesterol number) were provided for areas lacking laboratory services.

Both the **WHO PEN disease interventions** (13) and the **2020 HEARTS ‘D’ package** (14) recommendations for people living with Type 2 diabetes mellitus recommended statin treatment for people living with diabetes who are 40 years or older.

**WHO facility-based non-communicable disease performance indicators (2022)** (15) section C2 recommended reporting on the availability of statin medication. Another indicator titled ‘statin therapy among people with diabetes’, defined as ‘*proportion of people with diabetes (≥40 years old) who are receiving statin therapy based on WHO or national treatment guidelines*’. In concert with the WHO, the Member States endorsed global statin treatment coverage targets for people with diabetes, which set the goal that by 2030, 60% of people with diabetes above 40 years old should receive statins.

**WHO Model List of Essential Medicines (EML)** (16), last released in 2025, lists statins as the only ‘essential’ LLT ‘for use in high-risk patients’. Four dose intensities of simvastatin (5, 10, 20, and 40 mg) were listed with ‘black box’ equivalency for the statin molecules atorvastatin, fluvastatin, lovastatin, and pravastatin. Each statin molecule is associated with variable LDL-C lowering effect at the same dose, with atorvastatin being the most potent statin among those listed on the WHO EML.

**Figure 1:**
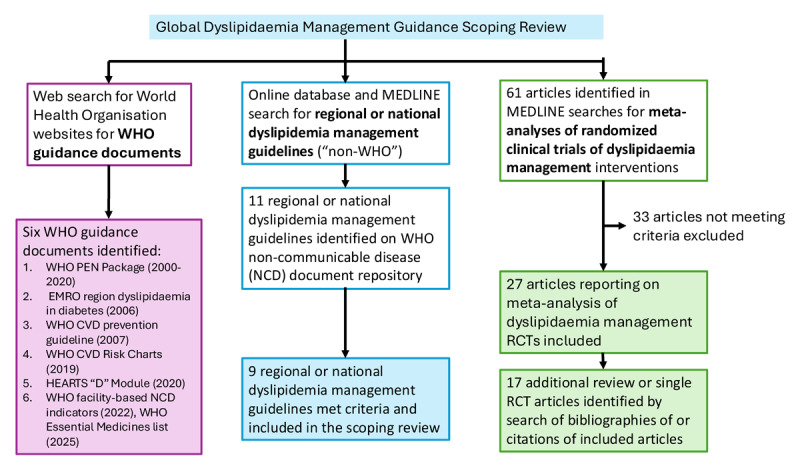
Flow chart for the global scoping review of dyslipidaemia management guidance.

In summary, six prior WHO guidance documents, including dyslipidaemia management recommendations, did not aim to be comprehensive and were limited in scope (Figure 1). These guidance documents focused on specific high-risk populations (in particular, people living with diabetes), general CVD prevention, or a limited aspect of dyslipidaemia management (e.g., defining essential LLT medications). WHO-recommended pharmacologic LLT options were limited to statins.

### Review of prior non-WHO dyslipidaemia management guidelines

A total of 11 non-WHO guidelines from across the globe were identified and reviewed (Table 1, **Appendix Table 3**). Most were published within the last five years; however, several older guidelines (released as early as 2006) were included in the interest of geographic inclusiveness. Some areas of the world were well-represented; however, others are not—particularly countries within Africa and Asia, in which identifying current guidelines proved challenging, perhaps in part because of our inability to access literature published locally and/or in languages not covered by this review exercise. Reasons for non-WHO guideline exclusion are described in detail in **Supplementary Appendix Table 3** and **Methods Appendix B**. Nine guidelines met the pre-defined inclusion criteria and were reviewed in detail (Table 1).

**Table 1:** Past non-WHO dyslipidaemia guidelines considered in this scoping review.


GEOGRAPHY	PUBLICATION YEAR	TITLE	INCLUSION STATUS/REASON FOR EXCLUSION	CITATIONS

**Regional dyslipidaemia management guidelines**				

Asia Pacific	2021	Asia Pacific Society of Cardiology Consensus Recommendations on Dyslipidaemia	**Exclude**. Reasons: management recommendations focused on CVD patients and other very high-risk groups, few primary prevention recommendations; authors report multiple potential conflicts of interest.	Koh (26)

Europe	2025	2025 Focused update of the 2019 ESC/EAS guidelines for the management of dyslipidaemias	Include	Mach (27)

Europe	2021	2021 ESC Guidelines on cardiovascular disease prevention in clinical practice	Include	Visseren (28)

Europe	2019	European Society of Cardiology/European Atherosclerosis Society Lipid Guidelines	Include	Mach (27)

**National dyslipidaemia management guidelines**				

China	2023	Chinese Guideline for Lipid Management	Include	Li (29)

India	2023	Lipid Association of India Dyslipidemia Guidelines	**Exclude**. Reasons: emphasis on diagnostic testing not available at primary care or local hospital levels; authors report multiple potential conflicts of interest.	Puri (30)

Sri Lanka	2021	Sri Lankan Ministry of Health Dyslipidaemia Management Guidelines	Include	Ministry of Health (31)

Brazil	2020	Brazil Ministry of Health Dyslipidaemia Management Guidelines	Include	Ministry of Health (6)

Kenya	2018	Kenya National Guidelines for Cardiovascular Diseases Management	Include	Ministry of Health (6)

USA	2018	AHA/ACC/AACVPR/AAPA/ABC/ACPM/ADA/AGS/APhA/ASPC/NLA/PCNA Guideline on the Management of Blood Cholesterol: A Report of the American College of Cardiology/American Heart Association Task Force on Clinical Practice Guidelines	Include	Grundy (32)

Egypt	2017	Egyptian Consensus of Dyslipidaemia Management	Include	Ministry of Health (6)

Mexico	2016	Mexican Dyslipidaemia Management Guidelines	Include	Mexican Institute for Social Security (6)


**Supplementary Appendix Tables 4a and 4b** detail the recommendations of the nine included regional or national guidelines. Summary observations were categorized into areas of consensus and non-consensus among these guidelines.

#### Areas of prior guideline consensus

- Most guidelines recommended non-fasting lipid testing (at least for initial screening) to test for dyslipidaemia status, for practical reasons
- All guidelines recommend lipid-lowering therapy without risk assessment for the highest risk groups (atherosclerotic cardiovascular disease (ASCVD) diagnosis, severely high cholesterol including familial hypercholesterolemia, diabetes, and other high-risk groups)
- Multivariable CVD risk assessment is a management component recommended by all guidelines covering primary ASCVD prevention in intermediate to high-risk groups without single risk factors determining high risk. Almost all national and regional guidelines recommend treatment for all patients with 10-year total ASCVD or CVD risk or CVD mortality risk >= 10% between the ages of 40–70 years.
- Most guidelines recommend high-intensity statin (e.g., atorvastatin 40–80 mg or rosuvastatin 20–40 mg) for patients with a history of clinical ASCVD or severely high cholesterol.
- Statin therapy is the universal standard first-line LLT; early initiation of other lipid-lowering therapies as add-on therapy (combined with statin) as part of early intensive LLT has been recommended for the highest risk patients. However, guidelines with emphasis on primary care settings and those for LMICs tend not to include recommendations for non-statin lipid-lowering therapy.
- The safety of statin therapy is universally recognized; however, all guidelines recommend suspending statin treatment during pregnancy

#### Areas of prior guideline non-consensus

- The CVD risk assessment tool recommended and the risk threshold to guide patient selection for lipid-lowering therapy vary by jurisdiction.
- Very few guidelines offered specific recommendations for how to assess ASCVD risk and select patients for lipid-lowering therapy when no laboratory testing is available.
- Outside of ASCVD and diabetes, high-risk conditions and categories indicating LLT varied among guidelines (e.g., variable inclusion of CKD, lipoprotein (a) [Lp(a)], hypertriglyceridemia, and HIV/other inflammatory conditions in the list of high-risk conditions).
- Lipid-lowering therapy goals (LDL-C goals) for primary and secondary prevention varied among guidelines.
- Use of non-statin lipid-lowering therapy varied, with guideline committees that represented high-income countries and regions recommending more novel therapies.
- Variable settings: Guideline clinical population focus varied, with some exclusively focused on primary care settings, others on specialized care settings, and yet others including both.
- Intensity and frequency of monitoring for LLT effectiveness and adverse effects varied among guidelines (passive versus active surveillance).
- Health economic considerations were almost universally absent from the guidelines reviewed. The European (ESC/EAS) 2019 guideline included a comprehensive review of LLT cost-effectiveness. The United States ACC/AHA 2018 guideline included a health economic evaluation, but it was dedicated to one new therapy: PCSK9 inhibitors. The cost-effectiveness of statins and most other LLTs for different CVD risk groups has not been integral to the formulation of dyslipidaemia guideline recommendations.
- Health equity issues were seldom discussed by the included guidelines. European guidelines do include social deprivation as a variable in CVD risk assessment equations.
- With the exception of the ESC/EAS CVD guidelines, social factors and health equity concerns were not included in guidelines. The guidelines of the United States, Sri Lanka, and Kenya stood out for including recommendations for overcoming guideline implementation barriers.

### Review of recent randomized controlled trials (RCTs) or systematic reviews of RCTs of dyslipidaemia treatments

Review of structured MEDLINE search results of 27 systematic reviews including meta-analyses of RCTs that had relevance in terms of supporting the priority areas for a dyslipidaemia management guideline, including LLT eligibility, LLT choice, and LLT targets, is provided in Table 2. An additional 17 reviews or single RCT reports identified in subsequent ‘snowballing’ searches supplemented the results of the main search strategy.

**Table 2:** Evidence mapping to dyslipidaemia management guideline priority topics: results of structured MEDLINE search.


GUIDELINE PRIORITY TOPIC	GUIDELINE SUBTOPIC	META-ANALYSES OF RCTS WITH CVD OUTCOMES IDENTIFIED IN STRUCTURED SEARCH	OTHER REVIEWS OR SINGLE TRIAL REPORTS

**Lipid-lowering treatment eligibility**	**Secondary prevention**	Pignone (33) CTT 2005 (34)	

	**Primary prevention: high risk defined by single risk factor**	Major (CKD) (36) Gami (diabetes) (37) Ponce (elderly) (35)	Hou (CKD) (38) Palmer(CKD) (39) Upadhyay (CKD) (40) Navaneethan (CKD) (41) Grinspoon (HIV) (42) De Vries (diabetes) (43) CTT 2008 (diabetes) (44) CTT 2019 (elderly) (45) Gencer (elderly) (46)

	**Primary prevention: other risk groups**	CTT 2012 (low CVD risk) (47) Karmali (CVD risk scores) (48) Pignone (all primary) (33)	

**Lipid-lowering treatment choice**	**Statin therapy intensity**	Hsu (49) Josan (50) Chan (51) Blasetto (52)	Smilde (FH) (53)

	**Non-statin therapies (alone or combined with statin)**	Hsu (49) Koskinas (54) Hosseni (55) Wu (56) Wang (57) Sayed (58) Sydhom (59) Navarese (60) Savarese (61) Du (62) Imran (63) Schmidt (64) Toth (65)	Perez de Isla (FH) (66)

**Lipid-lowering treatment goal**		Shepherd (67)	Jones (68) Rosenson (69)

**Lipid-lowering treatment monitoring and follow-up for efficacy or harms**		None	CTT 2019 (45) Chou (20) Collins (21) CTT 2010 (18)

**Equity**	**Gender stratified analysis**	CTT 2015 (19)	


Abbreviations: CTT: Cholesterol Lowering Trialists CKD: Chronic Kidney Disease CVD: Cardiovascular Disease FH: Familial Hypercholesterolemia HIV: Human Immunodeficiency Virus

Evidence informing other priority dyslipidaemia management topics was identified only in secondary review. These include RCT studies reporting on non-pharmacologic LLT (17), LLT safety (18,19,20,21), and timing of intensive LLT initiation (related to index CVD hospitalization) (22,23,24).

## Summary and Conclusions

This scoping review identified topics of major importance to be considered in the development of a global dyslipidaemia management guideline. These findings can be summarized as a series of questions, posed for each of the two major treatment groups—patients receiving LLT for primary or secondary prevention (Table 2):

- **Eligibility**: Which adults should receive lipid-lowering therapy (LLT) based on cardiovascular risk level and/or lipid profile?
- **Treatment Choice**: What LLT regimens (e.g., statin dose intensity, use of combination LLT) provide the best balance of efficacy, safety, and affordability?
- **Treatment Goals**: Should treatment aim for specific LDL-C targets or thresholds? If so, what targets are optimal in different populations and settings?
- **Monitoring and Follow-up**: What frequency and methods of lipid monitoring best support goal attainment and treatment adherence?
- **Equity and Cost-effectiveness**: What strategies are feasible, equitable, and cost-effective across different resource settings, particularly in LMICs?

A global dyslipidaemia management guideline should account for a variety of resource and care delivery contexts and address practical implementation issues. A practical guideline will address the question of fasting versus non-fasting lipid testing. Resource-constrained primary care settings and facilities lacking the capacity to test for lipid levels must necessarily adopt a streamlined approach using non-laboratory risk assessment to select higher risk patients for LLT, and concentrate on the availability and affordability of moderate-intensity statins. Evidence review should evaluate the role team-based dyslipdaemia management, and specifically the capacity of non-physicians to initiate and titrate lipid-lowering medicines. Practical implementation tools should be provided, including hypertension and diabetes treatment protocols that incorporate LLT for eligible hypertension or diabetes patients (25), and interventions to improve patient adherence to taking daily LLT medications.

The strength of this review is its global perspective and scope. Limitations include lack of a published scoping review protocol. Review of prior WHO and national and regional dyslipidaemia guidelines relied to an extent on desk review and key informant interviews, owing to lack of a comprehensive online repository for such guidance. This may introduce bias. Review of prior RCT evidence was limited to the MEDLINE database, for the formulation of a global guideline will require a more rigorous and systematic literature review of multiple databases. In addition, a global guideline for dyslipidaemia management must consider equity and cost-effectiveness to pursue maximal control of dyslipidaemia and CVD risk reduction in the context of constrained resources allocated for CVD prevention.

## Additional Files

The additional files for this article can be found as follows:

10.5334/gh.1506.s1 Supplementary file 1. Appendix Tables.

10.5334/gh.1506.s2 Supplementary file 2. Dyslipidaemia Scoping Review.

## Abbreviations

WHO: World Health Organization

CVD: cardiovascular disease

Tchol: total cholesterol

LDL: low-density lipoprotein cholesterol

HDL: high-density lipoprotein cholesterol

TG: triglycerides

Lp(a): lipoprotein (a)

Statin: hydroxymethylglutaryl-CoA (HMG CoA) reductase inhibitor
