# Supplementary material for: Global Guidance for Dyslipidaemia Management in Adults: A Scoping Review
Source: Glob Heart. 2025 Dec 16;20(1):111. doi: 10.5334/gh.1506 (PMC12716257; doi:10.5334/gh.1506)
Supplement: Supplementary file 2. — Dyslipidaemia Scoping Review. [file gh-20-1-1506-s2.pdf]

**Appendix Table 1. Dyslipidaemia definitions**

**Interpretation of cholesterol levels** (ACC/AHA 2018; ESC/EAS 2019)

| component | units | very low | low | ideal | high | severely high | alt units | very low | low | ideal | high | severely high |
|---|---|---|---|---|---|---|---|---|---|---|---|---|
| TC | mg/dl | | | 150 | 200 | 300 | mmol/L | | | 3.9 | 5.2 | 7.8 |
| LDL | mg/dl | 50 | 70 | 100 | 130 | 190 | mmol/L | 1.3 | 1.8 | 2.6 | 3.4 | 4.9 |
| TG | | | | 150 | 400 | 500 | mmol/L | | | 1.7 | 4.5 | 5.6 |
| Lp(a) | md/dl | | | | 50 | 180 | nmol/L | | | | | 430 |

TC = total cholesterol
LDL = low density lipoprotein cholesterol
TG = triglycerides
Lp(a) = lipoprotein (a)

| Lipid component | convert mg/dl to mmol/L, divide by | convert mmol/L to mg/dL, divide by | convert mg/dl to nm/L, multiply by |
|---|---|---|---|
| TC, LDL, HDL, non-HDL | 38.60 | 0.03 | |
| Triglycerides | 88.60 | 0.01 | |
| Lp(a) | | | 2.39 |

**Appendix Table 2. Criteria for prior non-WHO dyslipidaemia guideline inclusion and evaluation**

| Major Category | Subcategories |
|---|---|
| Timely | - Published within the prior 20 years (after 2005) |
| Evidence-based | - Methodology defined in manuscript<br>- Incudes systematic comprehensive literature review with standardized evidence-based grading criteria<br>- Guidelines reviewed by a central committee |
| Longevity and sustainability | - Guidelines updated periodically<br>- Stated planned follow up of guideline mentioned |
| Addresses key issues | - Measurement of serum cholesterol and other lipid components<br>- Criteria for the diagnosis of Dyslipidaemia, other indications for lipid-lowering therapy, and recommended follow up<br>- Defines optimal investigation for the diagnosis and management of Dyslipidaemia<br>- Assessment of overall cardiovascular risk and implications<br>- Addresses special treatment groups |
| Addresses broad audiences | - Multiple disciplines consulted (lipidologists, endocrinologists, cardiologists, primary care physicians, nurses, pharmacists, patients)<br>- Healthcare provider guidelines<br>- Includes ethnic/ racial considerations |
| Credible source | - National / organizational guidelines |
| Addresses conflict of interest | - Includes mention of conflicts of interest |
| Comprehensive | - Includes diagnostic and treatment algorithms |
| Published in major respected forum | - Published in print or online either in major journal or on organization's website |
| Stakeholder input and review | - Multidisciplinary review committee |

**Appendix Tables 3-5 are available as Microsoft Excel files.**

**Appendix Table 3. Inclusion/exclusion of full text articles of RCT evidence**

**Appendix Table 4. Inclusion of regional or national dyslipidaemia guidelines reviewed**

**Appendix Table 5. Detailed summary of regional or national dyslipidaemia guidelines**

**Methods Appendix A. PRISMA Extension for Scoping Reviews (PRISMA-ScR) reporting criteria checklist**

## Scoping Reviews (PRISMA-ScR) Checklist

| SECTION | ITEM | PRISMA-ScR CHECKLIST ITEM | REPORTED ON PAGE # |
|---|---|---|---|
| **TITLE** | | | |
| Title | 1 | Identify the report as a scoping review. | Title (title page) |
| **ABSTRACT** | | | |
| Structured summary | 2 | Provide a structured summary that includes (as applicable): background, objectives, eligibility criteria, sources of evidence, charting methods, results, and conclusions that relate to the review questions and objectives. | Abstract (page 2) |
| **INTRODUCTION** | | | |
| Rationale | 3 | Describe the rationale for the review in the context of what is already known. Explain why the review questions/objectives lend themselves to a scoping review approach. | Introduction (pages 3-5) |
| Objectives | 4 | Provide an explicit statement of the questions and objectives being addressed with reference to their key elements (e.g., population or participants, concepts, and context) or other relevant key elements used to conceptualize the review questions and/or objectives. | Introduction (page 5) |
| **METHODS** | | | |
| Protocol and registration | 5 | Indicate whether a review protocol exists; state if and where it can be accessed (e.g., a Web address); and if available, provide registration information, including the registration number. | No scoping review protocol was developed or registered |
| Eligibility criteria | 6 | Specify characteristics of the sources of evidence used as eligibility criteria (e.g., years considered, language, and publication status), and provide a rationale. | Selection criteria are described on pages 5-7, Figure 1 and Appendix Table 2 |
| Information sources* | 7 | Describe all information sources in the search (e.g., databases with dates of coverage and contact with authors to identify additional sources), as well as the date the most recent search was executed. | Information sources are described on pages 5-7 and Table 1 |
| Search | 8 | Present the full electronic search strategy for at least 1 database, including any limits used, such that it could be repeated. | Search strategies are described on pages 5-7 and MEDLINE search provided in Methods Appendix B |
| Selection of sources of evidence† | 9 | State the process for selecting sources of evidence (i.e., screening and eligibility) included in the scoping review. | Selection criteria are described on pages 5-7 and Appendix Table 2 |
| Data charting process‡ | 10 | Describe the methods of charting data from the included sources of evidence (e.g., calibrated forms or forms that have been tested by the team before their use, and whether data charting was done | Data charting of prior guidelines is shown in Appendix Tables 4 and 5 |

| SECTION | ITEM | PRISMA-ScR CHECKLIST ITEM | REPORTED ON PAGE # |
|---|---|---|---|
| | | independently or in duplicate) and any processes for obtaining and confirming data from investigators. | |
| Data items | 11 | List and define all variables for which data were sought and any assumptions and simplifications made. | Dyslipidaemia and related terms are described on pages 4 and 5 |
| Critical appraisal of individual sources of evidence§ | 12 | If done, provide a rationale for conducting a critical appraisal of included sources of evidence; describe the methods used and how this information was used in any data synthesis (if appropriate). | Inclusion and exclusion criteria are described on pages 5-7 and Appendix Tables 3,4, and 5. |
| Synthesis of results | 13 | Describe the methods of handling and summarizing the data that were charted. | Evidence was synthesized by one reviewer (AEM) and this process was overseen by other authors. |
| **RESULTS** | | | |
| Selection of sources of evidence | 14 | Give numbers of sources of evidence screened, assessed for eligibility, and included in the review, with reasons for exclusions at each stage, ideally using a flow diagram. | Selection results are described on pages 5-7 and Figure 1 |
| Characteristics of sources of evidence | 15 | For each source of evidence, present characteristics for which data were charted and provide the citations. | Information source characteristics are described on pages 5-7 and Appendix Tables 3-5 |
| Critical appraisal within sources of evidence | 16 | If done, present data on critical appraisal of included sources of evidence (see item 12). | Critical appraisal reported on pages 8-15, Table 1, and Table 2 |
| Results of individual sources of evidence | 17 | For each included source of evidence, present the relevant data that were charted that relate to the review questions and objectives. | Summary findings are reported in Table 1 and Table 2 |
| Synthesis of results | 18 | Summarize and/or present the charting results as they relate to the review questions and objectives. | Synthesis of results of the review of three information sources (WHO, regiona/national guidelines, RCTs) provided on pages 11-15 |
| **DISCUSSION** | | | |
| Summary of evidence | 19 | Summarize the main results (including an overview of concepts, themes, and types of evidence available), link to the review questions and objectives, and consider the relevance to key groups. | Summary of the evidence provided on page 15 |
| Limitations | 20 | Discuss the limitations of the scoping review process. | Limitations are described on page 16 |
| Conclusions | 21 | Provide a general interpretation of the results with respect to the review questions and objectives, as well as potential implications and/or next steps. | Conclusions reported on page 15 |
| **FUNDING** | | | |

| SECTION | ITEM | PRISMA-ScR CHECKLIST ITEM | REPORTED ON PAGE # |
|---|---|---|---|
| Funding | 22 | Describe sources of funding for the included sources of evidence, as well as sources of funding for the scoping review. Describe the role of the funders of the scoping review. | Role of the funding source reported on page 16-17 |

JBI = Joanna Briggs Institute; PRISMA-ScR = Preferred Reporting Items for Systematic reviews and Meta-Analyses extension for Scoping Reviews.

* Where *sources of evidence* (see second footnote) are compiled from, such as bibliographic databases, social media platforms, and Web sites.

† A more inclusive/heterogeneous term used to account for the different types of evidence or data sources (e.g., quantitative and/or qualitative research, expert opinion, and policy documents) that may be eligible in a scoping review as opposed to only studies. This is not to be confused with *information sources* (see first footnote).

‡ The frameworks by Arksey and O'Malley (6) and Levac and colleagues (7) and the JBI guidance (4, 5) refer to the process of data extraction in a scoping review as data charting.

§ The process of systematically examining research evidence to assess its validity, results, and relevance before using it to inform a decision. This term is used for items 12 and 19 instead of "risk of bias" (which is more applicable to systematic reviews of interventions) to include and acknowledge the various sources of evidence that may be used in a scoping review (e.g., quantitative and/or qualitative research, expert opinion, and policy document).

## Methods Appendix B. Appendix Methods

### Structured literature review method for identifying randomized controlled trials (RCTs) of dyslipidaemia management

The search strategy combined two structured key word searches with a structured MeSH term search. The results of these searches are reported in the manuscript and **Figure 1**.

1. ((((primary prevention) AND (lipid-lowering therapy)) AND (randomized controlled trials)) AND (systematic reviews)) AND (meta-analysis)

   Yield: 36 articles

2. ((((secondary prevention) AND (lipid-lowering therapy)) AND (randomized controlled trials)) AND (systematic reviews)) AND (meta-analysis)

   Yield: 19 articles

3. ("cardiovascular diseases/prevention and control"[MeSH Terms] OR "myocardial ischemia/prevention and control"[MeSH Terms] OR "myocardial infarction/prevention and control"[MeSH Terms] OR "stroke/prevention and control"[MeSH Terms]) AND "cholesterol, ldl/blood"[MeSH Terms] AND "treatment outcome"[MeSH Terms] AND "meta-analysis"[Publication Type]

   Yield: 16 articles

After removing duplicates, the total search yield was 61 unique articles (**see Figure 1**).

### Summaries of individual dyslipidaemia management guidelines reviewed

### Prior WHO Dyslipidaemia guidance

1. <u>WHO-HEARTS "D" Module (2020)</u>

   This WHO HEARTS module was released later than the original HEARTS package modules (released in 2017). The exclusive focus of this guideline is type 2 diabetes mellitus and does not cover other Dyslipidaemia treatment groups.
   Page 17: *"Statins are recommended for all people with type 2 diabetes 40 years old or older, but only if this does not negatively impact access to glucose-lowering and blood pressure-lowering medication."*
   Page 24: *"If this is not feasible, use statins in patients at highest risk of CVD events (e.g. patients with CVD, patients with nephropathy and/or patients with a high risk on a CVD risk prediction chart)."*
   No details provided regarding statin dose, treatment goals, or monitoring for treatment response of adverse effects.

Treatment protocols included in the HEARTS "D" module do not integrate the recommendation to initiate statin in patients with diabetes age 40 or above.
*Decision: Exclude; limited to guidance for people with diabetes*

2. EMRO 2006 Guideline for Dyslipidaemia in Diabetes
   As the title of this guideline suggests, this guideline is restricted to management of Dyslipidaemia in people with diabetes.
   Recommend only fasting lipid laboratory testing
   No CVD risk assessment component because focused on people with diabetes only.
   Multiple lipid-lowering therapy options described.
   No discussion of secondary prevention among people with a CVD diagnosis.
   Altogether, limited in scope because restricted to patients with diabetes; recommendation of only fasting lipid testing is practically limiting.
   Decision: Exclude; limited to guidance for people with diabetes

**Non-WHO regional and national Dyslipidaemia guidelines**

1. United States: ACC/AHA 2018
   Follow-on to 2013 ACC/AHA guideline
   Prior to 2013, it was US government sponsored/shift to ACC/AHA
   Multidisciplinary writing group
   Rigorous systematic reviews and evidence quality evaluations lead to systematic assignment of level of recommendation
   Screen all adults, but most recs for age 40+
   Major shift away from treatment based on lipid level to purely risk-based (except LDL>=190)
   Ten-year risk based on Pooled Cohort Equations (PCEs), mostly biological factors, did include Black, White, Other race/ethnicity. **It is expected that AHA PREVENT equations will replace the PCEs in future US guidelines*.
   LDL treatment goals only in ASCVD and other high-risk patients
   Statin emphasis, but novel treatments considered
   First ever use of economic evaluation of the case of PCSK9is to guide recommendations
   *Decision: Include*

2. Europe: ESC/EAS 2022
   Follow-on to the 2016 ESC/EAS guideline
   Cardiologist/lipid specialist writing group
   Rigorous systematic reviews and evidence quality evaluations lead to systematic assignment of level of recommendation
   Lipid screen and risk assess men 40+ and women 50+

Emphasis on 10-year fatal CVD risk assessed with Systematic Coronary Risk Estimation (SCORE) equations. Score equations are adapted to high and low risk regions of Europe, and in many cases to individual countries. In contrast with US, SCORE equations consider more social and mental health factors (social deprivation index, stress/anxiety/depression, physical inactivity)Unlike US guidelines, treatment is recommended based on a *cross-tabulation* of ten-year risk (very high risk, high risk, moderate risk, low risk) and LDL-C level; therefore more weight given to LDL-C level in ESC-EAS
Statin emphasis, but novel treatments considered
Comprehensive review of cost-effectiveness evaluations of non-pharm and pharm LLT
*Decision: Include*

3. 2021 Asia Pacific Society of Cardiology Dyslipidaemia Guideline

Gathered cardiovascular disease experts from across the Asia-Pacific Region; used a systematic review and consensus process for reviewing and approving recommendations.
The APSC guideline is limited to patients at relatively high risk, identified by Asia Pacific "CVD" system (ASCVD, diabetes, CKD, ischemic HF, microalbuminuria)
Due to high-risk focus, the guideline recommendations cover high intensity statin, ezetimibe, and PCSK9 inhibitors and diagnosis and management of familial hypercholesterolemia.
We found that the focus of this guideline was relatively narrow and not inclusive of many low-to-intermediate risk patients requiring Dyslipidaemia management in primary care settings.
Many potential conflicts of interest listed.
*Decision: Exclude; focus on hospital level management of patients with high CVD risk. Also, authors reported multiple potential conflicts of interest.*

4. 2023 Chinese guidelines for lipid management

Risk assessment is based on Chinese epidemiologic cohort studies and stratification differs from US/Europe, risk-enhancing factors for up-risking "moderate ASCVD risk" patients also different.
Recommend using moderate intensity statin in Chinese patients, concern for adverse effects with high intensity statin in this population
Recommend Xuezhikang (XZK) (Chinese Traditional Medicine) for lipid-lowering therapy when statin not tolerated, recommended before adding other non-statin LLT
Post-statin treatment monitoring includes more testing (liver enzymes and CK in addition to post-treatment LDL-C) and is more frequent compared with US/Europe (4-6 weeks post initiation, 3-6 months once stably treated)
*Decision: Include*

5. 2023 Lipid Association of India guidelines

This GL emphasizes that the unique epidemiology of ASCVD in India (South Asia) justifies a different approach for this population, prioritizing lifetime > 10 year ASCVD risk
Indians develop ASCVD about a decade earlier compared with Western populations (InterHEART, GBD Study) and half of CHD deaths in India occur before age 50 years
Inclusion of air pollution exposure as a high risk feature
Many potential conflicts of interest listed.
*Decision: Exclude; focus on hospital level management of patients with high CVD risk. Also, authors reported multiple potential conflicts of interest.*

6. 2016 Mexican Dyslipidaemia Management Guideline (Government of Mexico; CENETEC; IMSS)
   Recommend using the Mexico-specific Globorisk equation to estimate 10-yr CVD risk
   Recommend specific LDL-C treatment goals based on FH, diabetes, 10-year risk
   Method was to review several existing guidelines (USA, Australia)
   *Multiple Dyslipidaemia guidelines on government site—endorsed GL not clear*
   *Government publications—not peer reviewed*
   *Conflicts of interest and specialization of authors not stated*
   *Decision: Include*

7. 2017 Egyptian Consensus of Dyslipidaemia management
   Collaborative guideline from MoH Egypt NCD branch along with Egyptian Cardiology and Diabetes Task Force
   Essentially follow ESC/EAS guideline
   European SCORE risk charts for high risk regions
   Process not transparent, though basically relied on ESC/EAS guideline
   Supported by an unrestricted educational grant from AstraZeneca Egypt
   *Conflicts of interest and specialization of authors not stated*
   *Decision: Exclude*

8. 2018 Kenyan National Guidelines for Cardiovascular Diseases Management
   This is a practical set of guidelines for primary care-based CVD prevention measures, including management of Dyslipidaemia
   For risk assessment, the WHO-ISH risk charts for AFRO are recommended; 10-year risk stratified on 10-20, 20-30, and >=30% risk
   For the 10-20% ten-year risk category, lifestyle modification recommended first, and statin if cholesterol not at goal (“moderate reduction”) 3-6 months later; “moderate reduction” not defined.
   The only Dyslipidaemia treatments discussed are those available to primary care: lifestyle modification and statin treatment.
   Non-fasting lipid test acceptable, very high cholesterol defined for TC and LDL-C
   *Decision: Include*

9. 2021 Brazilian CLINICAL PROTOCOL AND THERAPEUTIC GUIDELINES FOR DYSLIPIDAEMIA: PREVENTION OF CARDIOVASCULAR EVENTS AND PANCREATITIS
   Systematic review
   Conflicts clear
   Government publication, indexed on WHO repository
   Use Framingham risk calculator, which is based on historical U.S. population data
   Treat diabetes + risk factors if male, age >=45, female, age >=55
   Framingham risk >=10%
   *Decision: Include*

10. 2021 Sri Lankan National Dyslipidaemia Guidelines for Primary Providers
   Expert panel (cardiology, endocrinology, nephrology)
   No systematic review
   No conflicts stated
   Government publication, not indexed on WHO repository
   Use WHO risk calculator for SE Asia region (Globorisk calculator)
   Treat diabetes, CKD, CVD risk >=20%, or TChol >-300 or LDL >=190
   Only recommend statins, no other LLT (maybe bc primary care GL)
   Government publication—not peer reviewed
   Conflicts of interest and specialization of authors not stated
   *Decision: Include*

11. 2017 Egyptian Consensus of Dyslipidaemia Management
   Expert panel consisting of cardiologists, academic general internists, and endocrinologists/lipidologists
   No systematic review, relied on European Atherosclerosis Society (EAS) guideline (see non-WHO guideline #2 above)
   No conflicts stated
   Guideline development supported by Astra Zeneca-Egypt
   Government publication, not indexed on WHO repository
   Use EAS calculator for high-risk European regions
   Treat diabetes, CKD, TChol >310, elevated EAS SCORE (Very high >10%, High 1-5%, Moderate >1%)
   Define LDL-C goals of <70mg/dl, <100 mg/dl, or <115 mg/dl for very high, high, moderate risk groups
   Discuss option of non-statin LLT (ezetimibe, cholestyramine, PCSK9i) if LDL-C goal not reached
   Government publication—"Under the supervision of the Ministry of Health non-communicable disease unit"
   Conflicts of interest not specifically stated
   *Decision: Include*
